# Supplementary material for: Towards unified AI-driven fracture mechanics: the extended deep energy method (XDEM)
Source: Nat Commun. 2026 Aug 15;17:8492. doi: 10.1038/s41467-026-76748-1 (PMC13484471; doi:10.1038/s41467-026-76748-1)
Supplement: Supplementary file 3 [file 41467_2026_76748_MOESM3_ESM.zip › pic/Results/Stress_intensity_factor/Mode1/a05_k1_comparison.pdf]

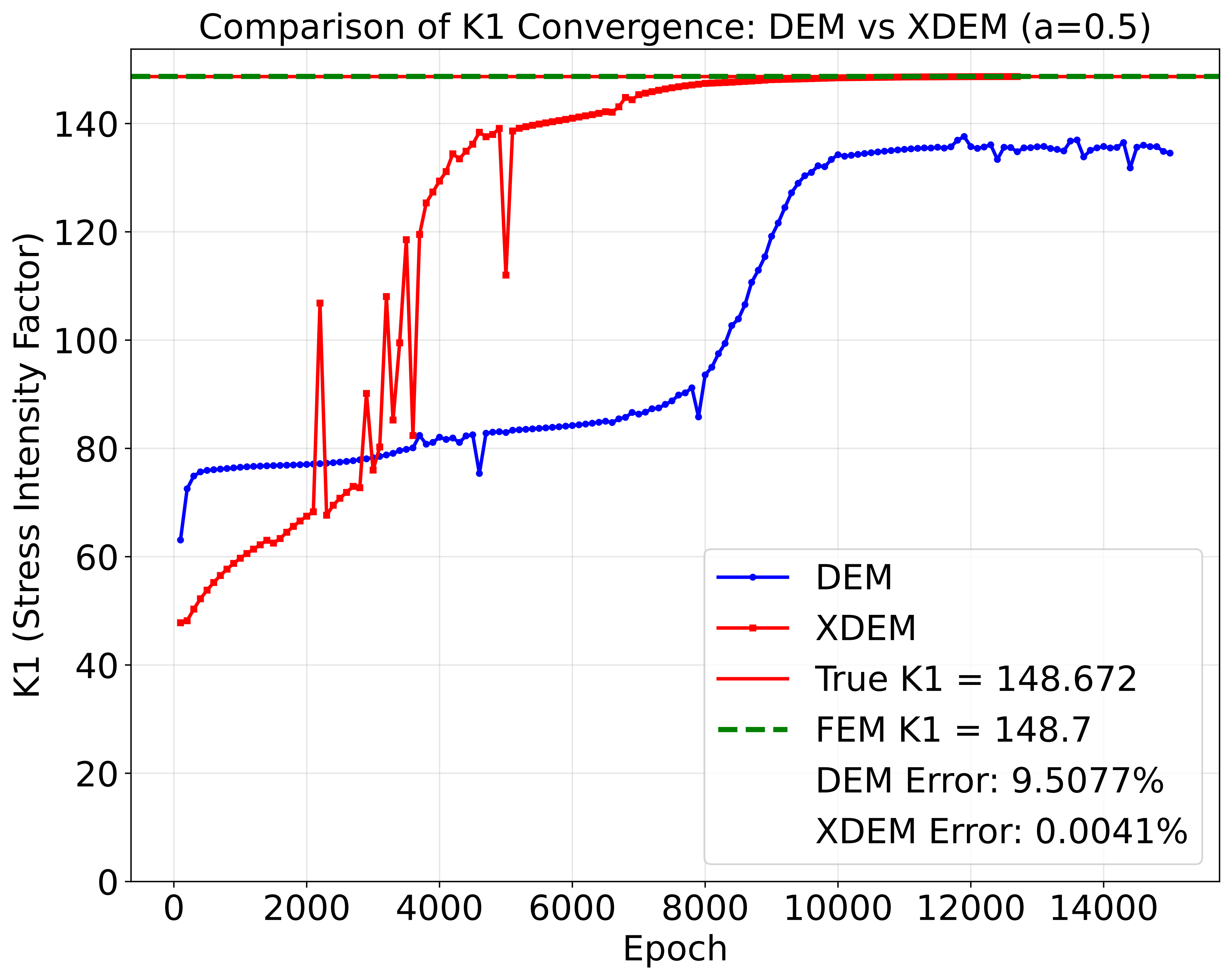
Comparison of K1 Convergence: DEM vs XDEM (a=0.5)
K1 (Stress Intensity Factor)
Epoch
0
20
40
60
80
100
120
140
0
2000
4000
6000
8000
10000
12000
14000
DEM
XDEM
True K1 = 148.672
FEM K1 = 148.7
DEM Error: 9.5077%
XDEM Error: 0.0041%
